# Exopolymer-Functionalized Nanoselenium from *Bacillus subtilis* SR41: Characterization, Monosaccharide Analysis and Free Radical Scavenging Ability

**DOI:** 10.3390/polym14173523

**Published:** 2022-08-27

**Authors:** Fengqin Wang, Man Du, Lixia Kai, Shuai Du, Weilian Hu, Yizhen Wang, Yuanzhi Cheng

**Affiliations:** 1 Key Laboratory of Animal Nutrition and Feed Science (Eastern of China), Ministry of Agriculture and Rural Affairs, Hangzhou 310058, China; 2 Key Laboratory of Animal Feed and Nutrition of Zhejiang Province, Hangzhou 310058, China; 3 School of Biological and Chemical Engineering, Zhejiang University of Science and Technology, Hangzhou 310035, China

**Keywords:** exopolymer-functionalized nanoselenium, monosaccharide composition analysis, free radical scavenging ability

## Abstract

To provide a safe and effective supplement of the essential trace element selenium, we focused on the biosynthesis of nanoselenium (SeNPs) via probiotics. A novel kind of exopolymer-functionalized nanoselenium (SeEPS), whose average size was 67.0 ± 0.6 nm, was produced by *Bacillus subtilis* SR41, whereas the control consisted of exopolymers without selenium (EPS). Chemical composition analysis, Fourier transform infrared (FTIR) spectroscopy and high-performance liquid chromatography (HPLC) confirmed that SeEPS and EPS shared similar polysaccharide characteristic groups, such as COO- and C=O, and contained not only 45.2–45.4% of sugars but also 23.5–24.7% of proteins and some lipids. Both SeEPS and EPS were primarily composed of mannose, amino glucose, ribose, glucose and galactose. Furthermore, to identify the biologically active component of SeEPS, three kinds of selenium particles with different stabilizers [Se(0), bovine serum albumin-Se and EPS-Se] were synthesized chemically, and their ability to scavenge free radicals in vitro was compared with that of SeEPS and EPS. The results revealed that EPS itself exhibited weak superoxide and hydroxyl radical scavenging abilities. Nevertheless, SeEPS had superior antioxidant properties compared to all other products, possibly due to the specific structure of SeNPs and exopolymers. Our results suggested that exopolymer-functionalized SeNPs with specific monosaccharide composition and structure could eventually find a potential application as an antioxidant.

## 1. Introduction

Selenium is an essential trace element that is present in the active sites of selenoenzymes such as glutathione peroxidase and thioredoxin reductase. Selenium has a narrow margin between its thresholds of functionality and toxicity; hence, the search for an appropriate and safe selenium addition form is ongoing [1].

Due to its unique size, surface properties, structure and chemical composition, nanoselenium, also known as selenium nanoparticles (SeNPs), has become the new focus of research in medical science [1,2], biology [3], botany [4] and animal husbandry [5]. By coating biological substances, such as proteins and polysaccharides, nanoselenium-functionalized biomaterials possess better beneficial effects than surface polymers or selenium alone [6,7]. The surface decoration of SeNPs with polymers enhanced their antioxidant or anticancer functions [8]. Meanwhile, these polymers participate in the reduction of inorganic selenium to SeNPs as size- and shape-regulating stabilizers or dispersants [9,10,11,12].

Different kinds of microbes, including bacteria [9,13], fungi [14], yeast [15] and algae [16], have been investigated for the synthesis of SeNPs through intracellular and extracellular processes. Bacteria are regarded as the most suitable biological factories because of their fast growth rate, low fermentation requirements, rich variety and ability to secrete a wide range of exopolymers [17,18,19]. Specifically, research indicates that *Bacillus* can tolerate and reduce a high dose of selenite into SeNPs, as well as produce a large number of exopolysaccharides with potent antioxidant and growth-promoting properties [20,21,22,23,24,25,26]. Previous research revealed exopolysaccharide-coated elemental SeNPs produced by *Bacillus paralicheniformis* (Se-BP) and investigated their antioxidative effects [27,28]. However, the size of Se-BP, which is 293 nm, means that it cannot be strictly classified as a nanomaterial, and its monosaccharide composition has not been identified. Consequently, there is still room for improvement in the prior research. These deficiencies motivated us to seek out alternatives.

The present study aimed to overcome these drawbacks by using an exopolymer-producing bacterial strain as the biological factory to biosynthesize SeNPs with high bioactivity and small particle size. Herein, we successfully prepared a novel exopolymer-functionalized nanoselenium (SeEPS) from *Bacillus subtilis* SR41. Subsequently, we (i) characterized the size, chemical composition, monosaccharide composition and surface features of SeEPS via chemical composition analysis, Fourier transform infrared (FTIR) spectroscopy and high-performance liquid chromatography (HPLC); (ii) prepared the exopolymers without selenium (EPS) and three kinds of selenium particles with different stabilizers [Se(0), bovine serum albumin (BSA)-Se and EPS-Se] for comparison; and (iii) investigated the antioxidant properties of SeEPS, EPS, Se(0), BSA-Se and EPS-Se on free radicals scavenging in vitro.

## 2. Materials and Methods

### 2.1. Chemicals and Reagents

Yeast extract and tryptone were purchased from OXOID (Hampshire, UK). Absolute alcohol, phosphate buffer powder (PBS), glucose, NaCl, K_2_HPO_4_ and MgSO_4_ were purchased from Sinopharm Chemical Reagent. (Sinopharm Chemical Reagent Co., Ltd., Shanghai, China). Sodium selenite, Vitamin C and BSA were purchased from Sigma-Aldrich (Sigma-Aldrich, St. Louis, MO, USA). The bicinchoninic acid (BCA) assay kit was purchased from KeyGEN (KeyGEN BioTECH, Nanjing, China). The total antioxidant capacity (T-AOC) detection kit was purchased from Beyotime (Beyotime Biotechnology, Shanghai, China). The Inhibition and Production of Superoxide Anion Assay Kit and Hydroxyl Free Radical Assay Kit were purchased from Nanjing Jiancheng (Nanjing Jiancheng Institute of Bioengineering Nanjing, China). Potassium bromide (KBr) was purchased from Aladdin (Aladdin Chemical Reagent Co., Ltd., Shanghai, China).

### 2.2. Bacteria Strains and Cultivation

*Bacillus subtilis* SR41, an exopolymer-producing bacterial strain, was identified and maintained in our laboratory and collected by the China General Microbiological Culture Collection Center (CGMCC No. 23536).

A single colony of *B. subtilis* SR41 was initially cultured in Lysogeny broth (tryptone, 1.0%, yeast extract, 0.5% and NaCl, 1.0%) at 37 °C overnight. Next, 4 mL of the seed fluid was inoculated into 100 mL cultivation culture (glucose, 2.0%, tryptone, 1.0%, yeast extract, 1.0%, K_2_HPO_4_, 0.1%, NaCl, 0.5% and MgSO_4_, 0.15%). The initial pH of the cultivation culture solution was adjusted to 7.0 with NaOH (0.1 mM). To obtain two kinds of exopolymers with or without selenium, 20 μL of sodium selenite working solution (0.578 M) or PBS was added to the cultures after fermentation for 6 h, respectively. The total incubation process was carried out on a rotary shaker (Sukun, Shanghai, China) at 37 °C and 200 rpm for 24 h.

### 2.3. Collection and Characterization of SeEPS and EPS

After cultivation, 300 mL of precooled absolute alcohol was added to the cultivation culture. The mixture was centrifuged at 4 °C, 5000× *g* for 10 min. After being washed twice with PBS, the suspension was ultrasonically crushed at 100 W, 10 s on/10 s off for 20 min using the SCIENTZ-IID ultrasonic cell breaker (Scientz Biotechnology, Ningbo, China). The average size of the nanoparticles was then measured using a Nano-ZS90 instrument (Malvern, Malvern, UK). The two kinds of prepared products were then lyophilized to produce dry powder of SeEPS (obtained from selenite-enriched culture) and EPS (obtained from culture without selenite).

The selenium concentration of SeEPS and EPS was measured via inductively coupled plasma mass spectrometry (Thermo Fisher, Shanghai, China). The total sugar and protein contents were measured using a phenol-sulfuric acid method and a BCA assay kit, respectively. The crude ash content was measured based on GB/T 6438-1992.

### 2.4. Preparation of Chemically Synthesized SeNPs

Three different kinds of chemical SeNPs were synthesized according to the method of Li et al. [29], with minor modifications. Using protein or exopolymers as a stabilizer, sodium selenite was reduced by vitamin C. In each case, 10 μL of sodium selenite working solution (0.578 M) was added to 20 mL of BSA solution (0.495 g/L) or EPS suspension (2.015 g/L). Then, 30 mL of a vitamin C solution (0.02 g/L) was cautiously and dropwise added under magnetic stirring. After 30 min, the pH of the solutions was adjusted to 10.0 with NaOH (0.1 mM). The BSA-Se and EPS-Se were then centrifuged at 10,000× *g* for 10 min and washed three times with double distilled water. To produce Se(0) as the negative control, 30 mL of vitamin C solution (0.02 g/L) was added dropwise into 20 mL of double-distilled water containing 10 mg/L sodium selenite without any protein or polymer. Characterizations of BSA-Se, EPS-Se and Se(0) were determined using the same technique as SeEPS and EPS.

### 2.5. Fourier Transform Infrared (FTIR) Spectral Analysis and Monosaccharide Composition Analysis

The functional groups of the SeEPS and EPS were detected by an FTIR spectrometer (Thermo Scientific, Waltham, MA, USA) in the frequency range of 4000 to 400 cm^−1^ using the KBr-disk method. Briefly, about 0.5 mg of the dry powder was mixed with 45 mg of KBr. The mixture was pelleted to 7 mm-thick under 12,000 kg using an automatic tablet press (Specas, Germany).

The monosaccharide composition of SeEPS and EPS was analyzed by HPLC according to the method we described before [30].

### 2.6. Determination of the Antioxidant Property

The superoxide radical scavenging effect of SeEPS, EPS, EPS-Se, BSA-Se and Se(0) was assessed according to the manufacturer’s instructions (Nanjing Jiancheng Institute of Bioengineering, Nanjing, China). Briefly, 50 μL of the sample suspensions at gradient concentrations (0.2, 0.5, 1.0, 2.0 and 5.0 mg/L) were mixed fully with reagent Ⅰ, reagent Ⅱ, reagent Ⅲ and reagent Ⅳ and were then heated in a 37 °C water bath for 40 min, followed by the addition of 2.0 mL of developer for 10 min. A total of 50 μL of the BSA solution (9.3 g/L) was tested as the control group. Absorbance was measured by the SpectraMaxM5 microplate reader (Molecular Devices, San Jose, CA, USA) with a 1 cm cuvette at a wavelength of 550 nm.

The hydroxyl radical scavenging activity of SeEPS, EPS, EPS-Se, BSA-Se and Se(0) was tested according to the manufacturer’s instructions (Nanjing Jiancheng Institute of Bioengineering, Nanjing, China). Briefly, 200 μL of sample solutions at different concentrations (0.2, 0.5, 1.0, 2.0 and 5.0 mg/L) were mixed with 200 μL of the substrate solution and 400 μL of reagent Ⅲ. After the reaction proceeded for 1 min at 37 °C, 2.0 mL of the developer was added immediately, and the tube was mixed and placed at room temperature for 20 min. A total of 50 μL of BSA solution (9.3 g/L) was tested as the control group. Absorbance was measured by the SpectraMaxM5 microplate reader with a 1 cm cuvette at a wavelength of 550 nm.

The total antioxidant capacities of SeEPS and EPS were measured as recommended according to the manufacturer’s instructions (Beyotime Biotechnology, Shanghai, China). Briefly, 200 μL of 2, 2′-azino-bis(3-ethylbenzothiazoline-6-sulfonic acid) diammonium salt (ABTS) working solution was reacted with 10 μL of the sample solution at different concentrations (0.2, 0.5, 1.0, 2.0 and 5.0 mg/L) for 6 min. Absorbance was measured by the SpectraMaxM5 microplate reader with a 1 cm cuvette at a wavelength of 734 nm. The standard curve was generated from different concentrations (0.15, 0.3, 0.6, 0.9, 1.2 and 1.5 mM) of Trolox (standard reagent); 50 μL of BSA solution (9.3 g/L) was tested as the control group.

### 2.7. Statistical Analysis

All data were expressed as the means ± standard deviation (SD) and performed using GraphPad Prism 8.0 (GraphPad Software, San Diego, CA, USA). All samples were prepared from three different batches of cultivation. The FTIR spectroscopic data were performed using OMNIC 9.2 (Thermo nicolet, Waltham, MA, USA).

## 3. Results

### 3.1. Components Determination of SeEPS and EPS

**Figure 1:**
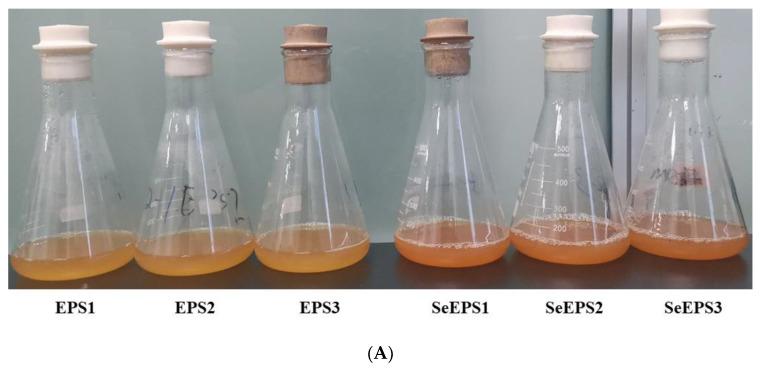

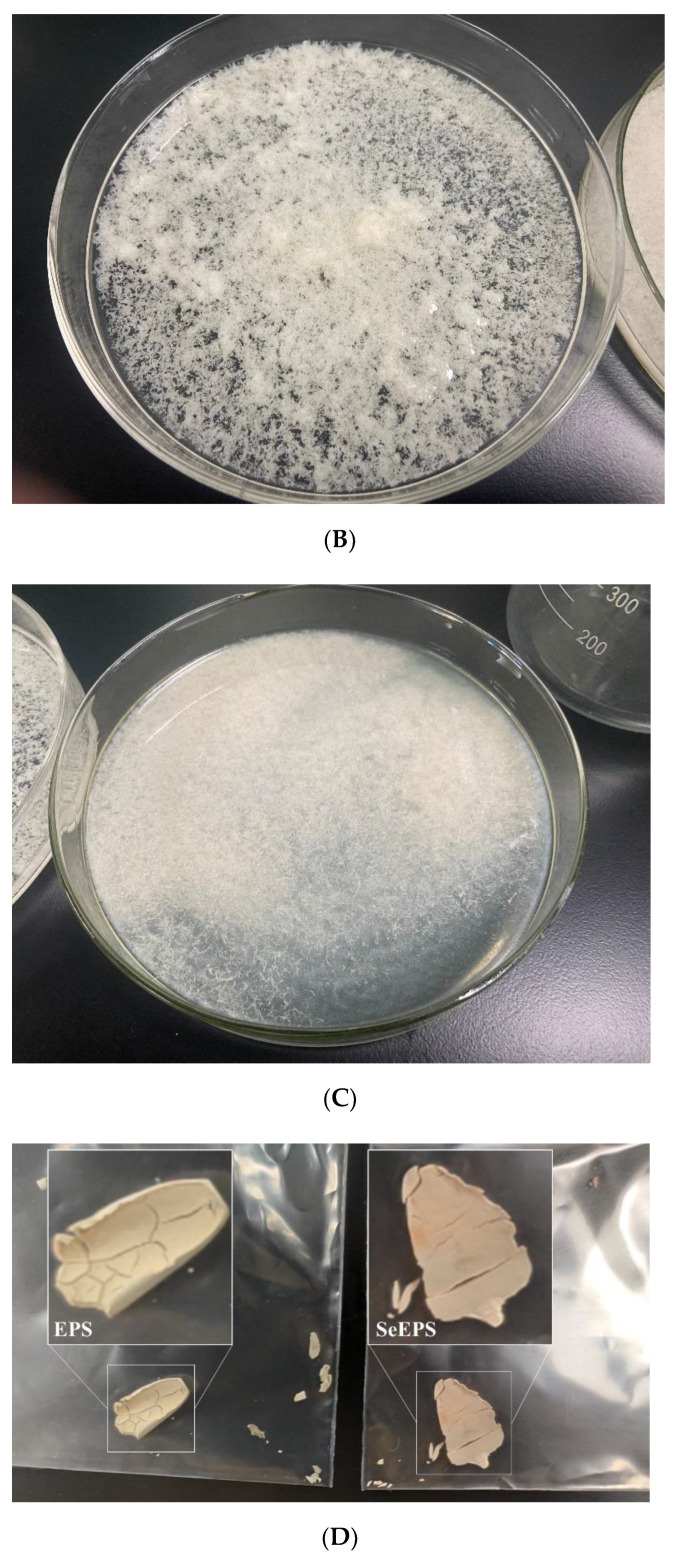
The whole process of sample preparation. (**A**) EPS and SeEPS after fermentation, (**B**) EPS after ethanol precipitation and centrifugation, (**C**) SeEPS after ethanol precipitation and centrifugation and (**D**) EPS and SeEPS after ultrasonication and lyophilization.

As shown in Figure 1A, after cultivating with the presence of 20 mg/L sodium selenite, the color of the *B. subtilis* SR41 broth changed to a light orange color. After the addition of ethanol, both EPS and SeEPS cultures exhibited flocculent precipitation, which represented exopolymers (Figure 1B,C). The dry matter contents of SeEPS and EPS were 37.8 ± 0.3 g/L and 40.3 ± 0.2 g/L, respectively. There was no significant difference between SeEPS and EPS in terms of the protein and sugar content. The total selenium content of SeEPS was 554.7 ± 9.5 mg/kg (Table 1).

In the following experiments, the concentrations of selenium are noted.

### 3.2. Characterization of SeEPS and EPS

**Figure 2:**
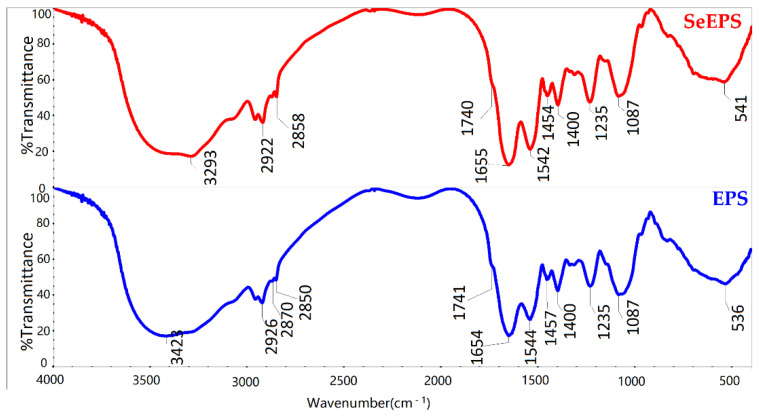
FTIR spectrum of SeEPS and EPS.

To investigate the surface compounds, SeEPS and EPS were lyophilized, and FTIR spectroscopy was used to determine their structures (Figure 2). The strong broad peak ranged from 3600 to 3200 cm^−1^ and corresponded to the stretching vibration peak of the O-H and N–H moieties. The CH2 asymmetric stretching vibration occurred at about 2930 cm^−1^. The symmetric CH2 vibration occurred at about 2870–2840 cm^−1^, and the CH2 scissoring vibration occurred at 1490–1440 cm^−1^. Additionally, 1740 cm^−1^ was the weak but typical absorption peak of ester C=O stretching vibrations [31]. A series of bands in the 1400–1000 cm^−1^ region was assigned to the stretching vibration of C-O, C-C and C-O-C [32,33]. The very typical amide I and amide II bands of proteins exhibited at about 1650 and 1540 cm^−1^ [34]. A weaker amide III band around 1235 cm^−1^ was sensitive to changes in the tertiary structure of the protein. Additionally, 1450 cm^−1^ corresponded to pyrrolidine ring vibrations of proline and hydroxyproline [35]. These data suggested that both SeEPS and EPS performed the same characteristic absorption peaks of the carbohydrate.

### 3.3. Monosaccharide Composition Analysis

**Figure 3:**
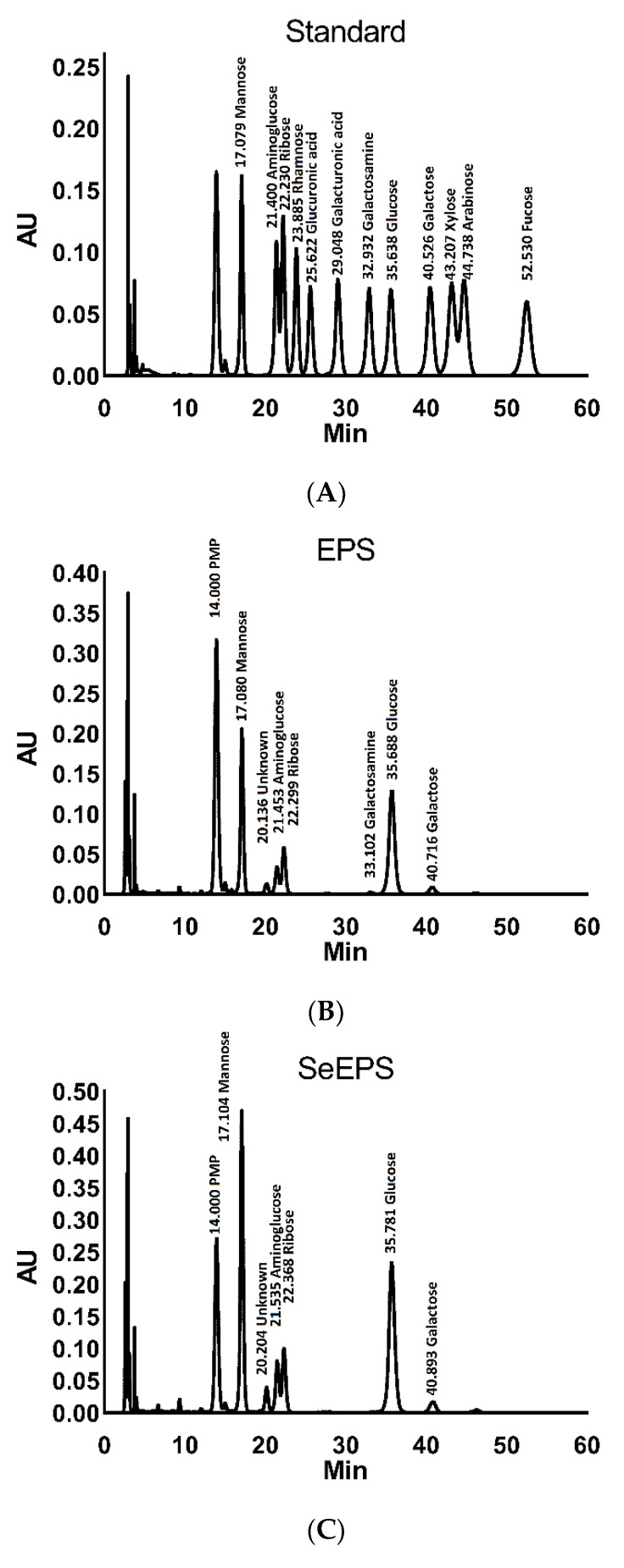

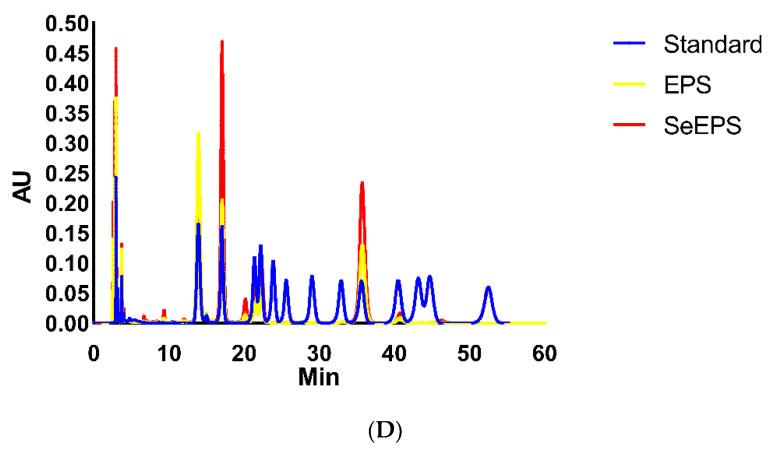
Monosaccharide composition analysis by HPLC. (**A**) peak spectrum for standards, (**B**) peak spectrum for EPS, (**C**) peak spectrum for SeEPS and (**D**) comparison among EPS, SeEPS and standards.

To further determine the monosaccharide composition in SeEPS and EPS, HPLC was utilized with 1-phenyl-3-methyl-5-pyrazolene precolumn derivatization (Figure 3). The results showed that SeEPS consisted of mannose, amino glucose, ribose, galactosamine, glucose and galactose in the following molar ratios: 34.9 ± 2.8%: 8.8 ± 1.2%: 10.1 ± 0.3%: 0.2 ± 0.1%: 37.8 ± 0.8%: 2.8 ± 0.3% (Table 2). Both SeEPS and EPS exhibited similar monosaccharide types with minor differences in terms of monosaccharide content.

### 3.4. Preparation and Characterization of EPS-Se, BSA-Se and Se(0)

To determine which component or specific structure of SeEPS played a decisive role in the biological function, three types of chemical SeNPs were synthesized to serve as the comparison; their selenium, protein and sugar contents were designed to match those of SeEPS.

As shown in Table 3, the differences in selenium particle size are a result of the composition and synthesis processes. SeEPS and BSA-Se were comparable in size, selenium and protein content, but BSA-Se lacked sugar. SeEPS and EPS-Se had nearly identical contents, but the average size of EPS-Se was nine times larger than that of SeEPS. Moreover, Se(0) was produced directly without protein or sugar as a stabilizer, with a size of 762 ± 18 nm.

### 3.5. Free Radical Scavenging Activities

**Figure 4:**
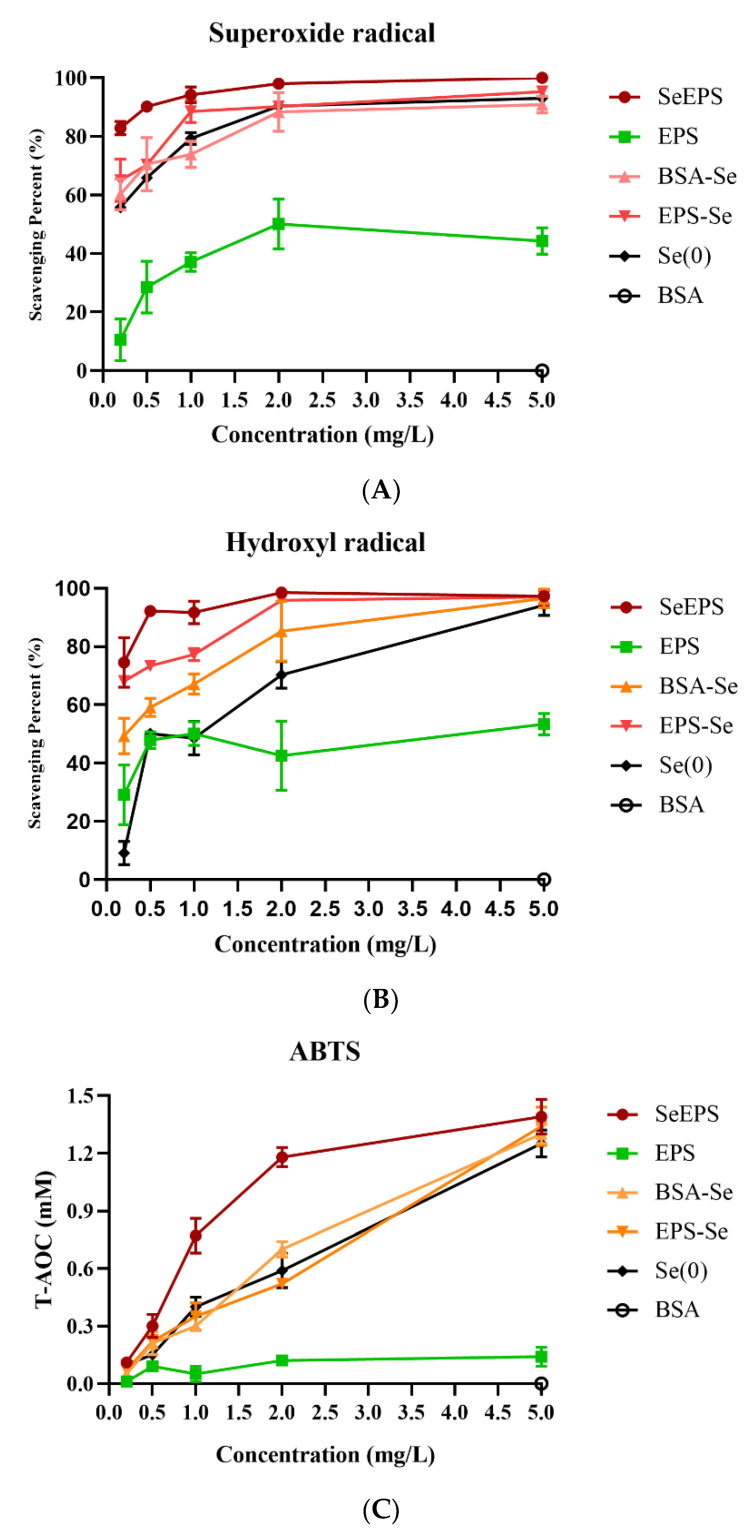
Free radical scavenging activities of SeEPS, EPS, EPS-Se, BSA-Se and Se(0): (**A**) scavenging activity of superoxide radical, (**B**) scavenging activity of hydroxyl radical and (**C**) scavenging activity of ABTS radical. BSA is used as a negative control.

As shown in Figure 4, all samples were able to scavenge three different kinds of free radicals in a dose-dependent manner, with SeEPS exhibiting the highest scavenging ability at concentrations between 0.25 and 2 mg/mL. At a concentration of 5 mg/mL, SeEPS, EPS-Se, BSA-Se and Se(0) almost completely eliminated free radicals. Additionally, when the sample concentration was below 0.25 mg/mL, we found that the results were not repeatable (not shown in the figure), which could be attributed to the low solubility and incomplete dispersion of SeEPS, EPS-Se and Se(0) at low concentrations.

## 4. Discussion

Selenium is one of the critical trace elements necessary to maintain growth and various biochemical and physiological functions in mammals by forming an integral part of numerous selenoenzymes and selenoproteins. Numerous studies have demonstrated that selenium deficiency is associated with oxidative stress or the excessive production of reactive oxygen species (ROS) [36,37,38]. As the toxic dose of selenium is slightly higher than the requirement, it is essential to select a selenium addition form with high efficiency and safety [1,39]. Sodium selenite, selenium-enriched yeast, DL-selenomethionine and SeNPs are common selenium supplements [9]. Typically, SeNPs have already been proposed for a variety of biomedical applications due to their antioxidant properties and distinct behavior relative to other selenospecies [13,40]. Moreover, SeNPs have distinct chemical and physical properties because of their large surface-to-volume ratio, high surface energy and spatial limitation [24,41]. Based on our prior research, we were able to successfully prepare a novel form of SeNPs with a size of 67.0 ± 0.6 nm.

Previously, SeNPs were synthesized by chemical methods, the principle of which was to use macromolecular substances as dispersants, such as chitosan [40,41], BSA [42], exopolysaccharide xanthan gum [43], polyvinylpyrrolidone C15 [44] and epigallocatechin-3-gallate [45], to convert inorganic selenium into elemental selenium by adding reducing agents such as vitamin C and GSH. The sizes of chemically synthesized SeNPs were irregular and varied from 20 to 50,000 nm [46]. To overcome these drawbacks, more attention has been paid to selenite-tolerated bacteria. *Bacillus licheniformis* [47], *Enterobacter cloacae* [12], *Lactobacillus casei* [48] and *Methylococcus capsulatus* [49] have been shown to effectively detoxify selenite and synthesize SeNPs of 50–200 nm. By coating the exopolymers secreted during reduction, SeNPs were dispersed and functionalized to combine the properties of both polymers and nanoparticles [50]. Dobias et al. revealed that the binding protein AdhP in *Escherichia coli* played a key role in controlling the morphology and particle size of SeNPs [11]. In this study, EPS and SeEPS produced by *Bacillus subtilis* SR41 exhibited the typical characteristics of an exopolymer after ethanol precipitation (Figure 1B,C), which is consistent with previously reported studies [8,17]. Furthermore, the FTIR and HPLC results confirmed that both EPS and SeEPS possessed functional groups characteristic of polysaccharides. As the processes of bacterial proliferation, inorganic selenium reduction and polymers synthesis all required energy consumption; bacteria would preferentially ensure their growth and reduced the inorganic selenium, for the total nutrients of the culture medium were consistent [28]. Interestingly, *B.subtilis* SR41 completely reduced 20 mg/L sodium selenite in only 24 h, and the output of SeEPS (37.8 ± 0.3 g/L) was slightly less than that of EPS (40.3 ± 0.2 g/L). HPLC analysis also supported the evidence that glucose, which was the only monosaccharide source in the culture medium, retained higher proportions in SeEPS than those in EPS (Figure 3). Meanwhile, EPS had 0.22% galactosamine, which was not detected in SeEPS, indicating that the polymer secretion ability of *B.subtilis* SR41 might be inhibited when inorganic selenium exists (Table 2). Further experiments were needed to explore whether *B.subtilis* SR41 had the ability to generate SeEPS when adding a high concentration of sodium selenite.

Multiple studies have demonstrated that the exopolymer-functionalized SeNPs possess superior biological functions compared to either exopolymers or selenium [6,51], so SeEPS may have a powerful antioxidant effect against ROS and oxidative stress. To investigate the antioxidant property of SeEPS, the ability of three kinds of free radicals to be scavenged in vitro was examined. The superoxide anion radical, created from the mitochondrial electron transport system, was considered as an initial radical to generate other cell-damaging free radicals [52]. The hydroxyl radical has the potential to degrade DNA and cause cell damage [53]. ABTS, which was generated by oxidants, was widely used to evaluate the T-AOC [54]. To determine the dose-dependent effect, the concentrations of samples were assessed at the gradient. As described above (Figure 4), SeEPS exhibited the greatest antioxidant activity at the concentration range of 0.25 to 2 mg/mL. Comparing the radical scavenging capabilities of Se(0), EPS-Se and BSA-Se revealed that, while EPS could partially scavenge free radicals, SeNPs played a more important role in the antioxidant process than polymers. Nevertheless, contrary to our previous hypothesis, the particle sizes of different products did not influence the results, whereas others believed that particles with a median size of 5–500 nm could be considered nanomaterials [55].

We believed that the high biological activity of SeEPS was due to the specific structure between exopolymers and SeNPs, and these results corresponded with a study that focused on SeNPs in combination with *Dictyophora indusiata* polysaccharide [8]. According to our previous study, when sodium selenite was present in the fermentation medium, a large number of reticular organic mosaic particles appeared outside the cell [9]. This phenomenon may be because inorganic selenium stimulates bacteria to produce selenium reduction-related proteins [56]. Ullah et al. hypothesized that the functional groups of selenium nanoparticles produced by *Bacillus Subtilis* BSN313 could be responsible for the reduction of sodium selenite [24]. Using FTIR spectroscopy, we determined that, regardless of whether inorganic selenium was present during fermentation, there was no significant difference in the functional groups of the products, since the FTIR spectrum curves of SeEPS and EPS were highly coincident (Figure 2).

## 5. Conclusions

The exopolymer-functionalized SeNPs from *Bacillus subtilis* SR41 were successfully produced. The average size of the nanoparticles in the SeEPS was 67.0 ± 0.6 nm. SeEPS and EPS shared similar polysaccharide characteristic groups, such as COO- and C = O, and contained not only 45.2–45.4% of sugars but also 23.5–24.7% of proteins and some lipids. HPLC results showed that SeEPS consisted of mannose, amino glucose, ribose, galactosamine, glucose and galactose in the following molar ratios: 34.9 ± 2.8%: 8.8 ± 1.2%: 10.1 ± 0.3%: 0.2 ± 0.1%: 37.8 ± 0.8%: 2.8 ± 0.3%. Additionally, compared with Se(0), BSA-Se and EPS-Se, which were all prepared through chemical synthesis with different stabilizers, SeEPS exhibited the greatest ability to scavenge free radicals. This study suggested that SeEPS could be a suitable selenium supplement. Further research is required to determine the biological function of SeEPS in the cell models and in vivo.

## Figures and Tables

**Table 1:** Chemical composition of SeEPS and EPS.

Item	SeEPS	EPS
Dry matter (g/L)	37.8 ± 0.3	40.3 ± 0.2
Protein contents (%)	24.7 ± 1.1	23.5 ± 1.7
Sugar contents (%)	45.2 ± 0.1	45.4 ± 0.1
Ash content (%)	8.4 ± 0.1	8.1 ± 0.2
Se contents (mg/kg)	554.7 ± 9.5	0

Data are shown as mean ± SD, *n* = 3.

**Table 2:** Monosaccharide composition of SeEPS and EPS.

Item	SeEPS	EPS
Mannose	34.9 ± 2.8%	34.2 ± 2.8%
Amino glucose	8.8 ± 1.2%	7.7 ± 1.0%
Ribose	10.1 ± 0.3%	11.1 ± 1.1%
Galactosamine	0.2 ± 0.1%	-
Glucose	37.8 ± 0.8%	35.4 ± 4.7%
Galactose	2.8 ± 0.3%	6.9 ± 5.6%
Unknown	4.3 ± 0.7%	3.1 ± 0.5%

Contents were calculated according to the retention time. Data are shown as mean ± SD, *n* = 3.

**Table 3:** Characterization of SeEPS, EPS, BSA-Se, EPS-Se and Se(0).

Item	Biogenic Products by SR41		Chemically Synthesized Products		
	SeEPS	EPS	BSA-Se Solution	EPS-Se Suspension	Se(0) Suspension
Selenium contents (mg/L)	20.1 ± 0.3	0	19.9 ± 0.2	20.0 ± 0.1	20.0 ± 0.2
Protein contents (g/L)	9.3 ± 0.1	9.3 ± 0.1	9.2 ± 0.2	9.3 ± 0.0	0
Sugar contents (g/L)	17.5± 0.3	18.1 ± 0.1	0	18.1± 0.2	0
Average size of nanoselenium (nm)	67.0 ± 0.6	-	56.8 ± 0.2	635 ± 21	762 ± 18

Contents were calculated according to the retention time. Data are shown as mean ± SD, *n* = 3.

## Data Availability

All data used during the study appear in the submitted article.
